# Post-Cancer Treatment Reflections by Patients Concerning the Provisions and Support Required for a Prehabilitation Programme

**DOI:** 10.1007/s00268-023-07170-7

**Published:** 2023-09-12

**Authors:** Amrita Kaur Jandu, Arpanun Nitayamekin, Josh Stevenson, Martin Beed, Ravinder S. Vohra, Vincent G. Wilson, Dileep N. Lobo

**Affiliations:** 1 School of Life Sciences, University of Nottingham, Queen’s Medical Centre, Nottingham, UK; 2 The Notts County Foundation CARE Programme, Portland Leisure Centre, Nottingham, UK; 3 Department of Anaesthesia, Nottingham University Hospitals NHS Trust, City Hospital Campus, Nottingham, UK; 4 Gastrointestinal Surgery, Nottingham Digestive Diseases Centre and National Institute for Health Research Nottingham Biomedical Research Centre, Nottingham University Hospitals NHS Trust and University of Nottingham, Queen’s Medical Centre, Nottingham, NG7 2UH UK; 5 Trent Oesophago-Gastric Unit, Nottingham University Hospitals NHS Trust, City Hospital Campus, Nottingham, UK; 6 MRC Versus Arthritis Centre for Musculoskeletal Ageing Research, School of Life Sciences, University of Nottingham, Queen’s Medical Centre, Nottingham, UK

## Abstract

**Background:**

Evidence suggests that physical fitness interventions, mental health support and nutritional advice before surgery (prehabilitation) could reduce hospital stay and improve quality of life of patients with cancer. In this study we captured the opinions of a group of patients with cancer undergoing these interventions after treatment to discover what a prehabilitation programme should encompass.

**Methods:**

Patients from the Cancer and Rehabilitation Exercise (CARE) programme based in Nottingham took part in a 26-point online questionnaire about the design of prehabilitation programmes.

**Results:**

The questionnaire was completed over a 2-week period in December 2021 by 54 patients from the CARE programme. Their responses were as follows: 44 (81.5%) participants would have participated in prehabilitation had it been available to them and 28 (51.9%) ranked physical exercise as the most important component. Forty (74.1%) participants believed the counselling aspect of prehabilitation would have contributed to a successful outcome and 35 (64.8%) thought dietary advice would have benefitted them before surgery. Thirty-one (57.4%) participants preferred the programme to take place in a fitness centre, rather than at home or hospital and 43 (79.6%) would have liked to have known about prehabilitation from their doctor at the time of diagnosis.

**Conclusions:**

Patients are interested in prehabilitation to become more physically fit and mentally prepared for surgery. They expressed the need for a focus on physical exercise, counselling to improve mental health and personalised nutritional advice. Tailoring a prehabilitation programme, with input from patients, could contribute to improving patient outcomes following cancer treatments.

**Supplementary Information:**

The online version contains supplementary material available at 10.1007/s00268-023-07170-7.

## Introduction

Prehabilitation comprises a multi-modal approach directed towards patient care that could encompass preoperative physical exercise, psychological support and nutritional optimisation. The intention is to enhance an individual’s functional capacity to withstand the stressors of major surgery, thereby potentially accelerating postoperative recovery and improving outcomes [1–3]. This is particularly important for high-risk and frail patients, who make up a significant proportion of hospital admissions for cancer treatment and surgery [4, 5], since they are more prone to experiencing adverse effects and complications that can reduce quality of life in the long-term. The concept of prehabilitation is not just for patients undergoing major surgery, but could also be applied to those undergoing non-surgical treatment for cancer [6, 7].

It is recognised that the preoperative period represents a ‘teachable moment’ in healthcare as individuals may be more receptive to instituting behavioural changes to their lifestyle. Patients show more confidence in making key changes in the period leading up to surgery, particularly related to weight management and lowering alcohol consumption, as they recognise that these are likely to yield greater benefit after treatment [8].

However, many prehabilitation protocols have been devised by healthcare professionals without necessarily seeking the views of patients on what would be acceptable. Furthermore, many regimens have largely involved unsupervised activities at home, principally for convenience, with the net outcome that compliance has been variable [3, 9]. This may account for why, despite the theoretical benefits, the outcome of randomised clinical trials and meta-analyses on prehabilitation have been variable [3, 10–13].

Other barriers to the successful implementation of a prehabilitation programme include psychosocial factors (e.g. hopelessness, overwhelming fear, lack of motivation), physical issues (e.g. neuropathy) and symptomatic factors (e.g. nausea, pain). In addition, the optimal length of a successful prehabilitation programme remains uncertain; some studies suggest 6 weeks, whereas others recommend 8–12 weeks, but this may not be possible due to the urgency of some procedures for cancer [3]. While a few studies have canvassed patients’ views on the suitability, desirability and feasibility of prehabilitation [14–18], some of these have been timed when the patients are also preoccupied with the diagnosis of cancer and the immediate prospect of major surgery [18].

Clearly, patient ‘buy-in’ is essential for any prehabilitation programme to be successful as compliance is a key factor, so it is crucial that we better understand the patients’ perspectives on their treatment journey. In Nottingham, the 12-week Cancer and Rehabilitation Exercise (CARE) programme run by the Notts County Foundation (official charity of Notts County Football Club) in partnership with Macmillan Cancer support helps with rehabilitation of patients who have completed their treatment for cancer. We engaged this cohort of patients to perform a questionnaire study on the optimal requirements for a prehabilitation programme. Crucially, the participants had knowledge of the treatment pathway for cancer and; therefore, potentially had greater insights into what would be most helpful and what is likely to work.

## Methods

### Study design and participants

This questionnaire study was performed on participants from the Notts County Foundation CARE programme who had undergone rehabilitation after completing both surgical and non-surgical treatment for cancer and were in remission at the time of study. Sessions take place in community-based gyms in Nottingham, Newark and Mansfield (roughly 15 miles apart) and aim to provide patients with physical exercise benefits as well as a social environment during this difficult period of their lives. In December 2021, the CARE Programme database held email details of 213 ex-patients and each was contacted by one of the investigators (JS) highlighting the study, together with information links about prehabilitation. Participation was voluntary and anonymous. A video was produced to advertise the questionnaire and introduce the potential participants to the concept of prehabilitation and its potential benefits. The link (https://www.youtube.com/watch?v=8PnphYPYYJk) was open for two weeks in December 2021 and during this time potential participants had the opportunity to speak to investigators (AN and AJ). Participants were contacted only once, and reminders were not sent.

### Questionnaire design

A 26-point questionnaire (online appendix 1) was created on Google Forms to ask participants about their own personal rehabilitation experiences and their thoughts on a hypothetical prehabilitation programme. These were partly framed by the work of Gillis and colleagues [16] who identified themes related to preparation for surgery, emotional needs and general support. The questions were also based on previous work undertaken at the University of Nottingham [19, 20]. Questions asked about frequency and location of prehabilitation sessions and mostly took a multiple choice or ranking format. An open-ended question, where participants could input free text regarding ideas about future prehabilitation programmes, was also included.

### Data analysis

Data from responses collected in Google Forms were inputted into Microsoft Excel where graphs were generated. Thematic analysis was performed by AJ and AN. All text responses were combined onto one document and actively reread several times to gain full understanding. After being familiar with the responses, initial codes from the data were generated next to texts that were insightful for the design of prehabilitation programme. All responses were given full and equal attention to ensure that no codes were missed. The different codes were sorted and combined into potential subthemes and themes. A thematic map was generated to visualise the relationship between the codes, forming subthemes and themes. They were checked back against each other and to the original responses, ensuring that they matched. These were then summarised into short coded themes based on ‘communication’, ‘diet/nutrition’ and ‘support’ and analysed based on frequency [21, 22].

### Ethics and consent

Ethical approval was obtained from the University of Nottingham, School of Life Sciences Research Ethics Committee (B081121VW) prior to inviting participants. Informed consent was obtained after providing information detailed previously. Information about how the data would be used was also offered. Participants could only proceed to the questionnaire if they ticked the consent box.

## Results

Fifty-four patients held on the CARE database (December 2021), who had undergone rehabilitation after treatment for cancer, agreed to participate in the study. Table 1 displays the demographics for the cohort, with a greater proportion of women than men, and surgery involved for 46 (85.2%) participants. The average wait time between diagnosis and surgery was 8 weeks, but for approximately 10% of respondents this was completed within 2 weeks. Eight patients (14.8%) did not receive surgical treatment. The adherence rate of the respondents for the CARE programme was high: all participants attended ≥ 6 of the 12 weekly scheduled sessions, with 31 (57.4%) missing ≤ 2 sessions. Furthermore, 29 (53.7%) respondents indicated that they possessed exercise equipment at home, with another 8 (14.8%) considering possible purchase of equipment at the time of the survey.

**Table 1:** Demographics of the participants

	*n*	%
*Sex*		
Female	33	61.1
Male	21	38.9
*Age (years)*		
Mean	61.2	
Range	33–78	
*Types of cancer*		
Breast	20	37.0
Prostate	8	14.8
Haematological	6	11.1
Bowel	6	11.1
Oesophageal	5	9.26
Other	11	20.4
*Types of treatment*		
Surgery	46	85.2
Chemotherapy	37	68.5
Radiotherapy	33	61.2
Hormone therapy	19	35.2
Other	4	7.40
*Waiting time between diagnosis and surgery*		
1–2 weeks	6	11.1
3–8 weeks	24	44.4
> 8 weeks	16	29.6
Did not have surgery	8	14.8

### Interest in prehabilitation

**Fig. 1:**
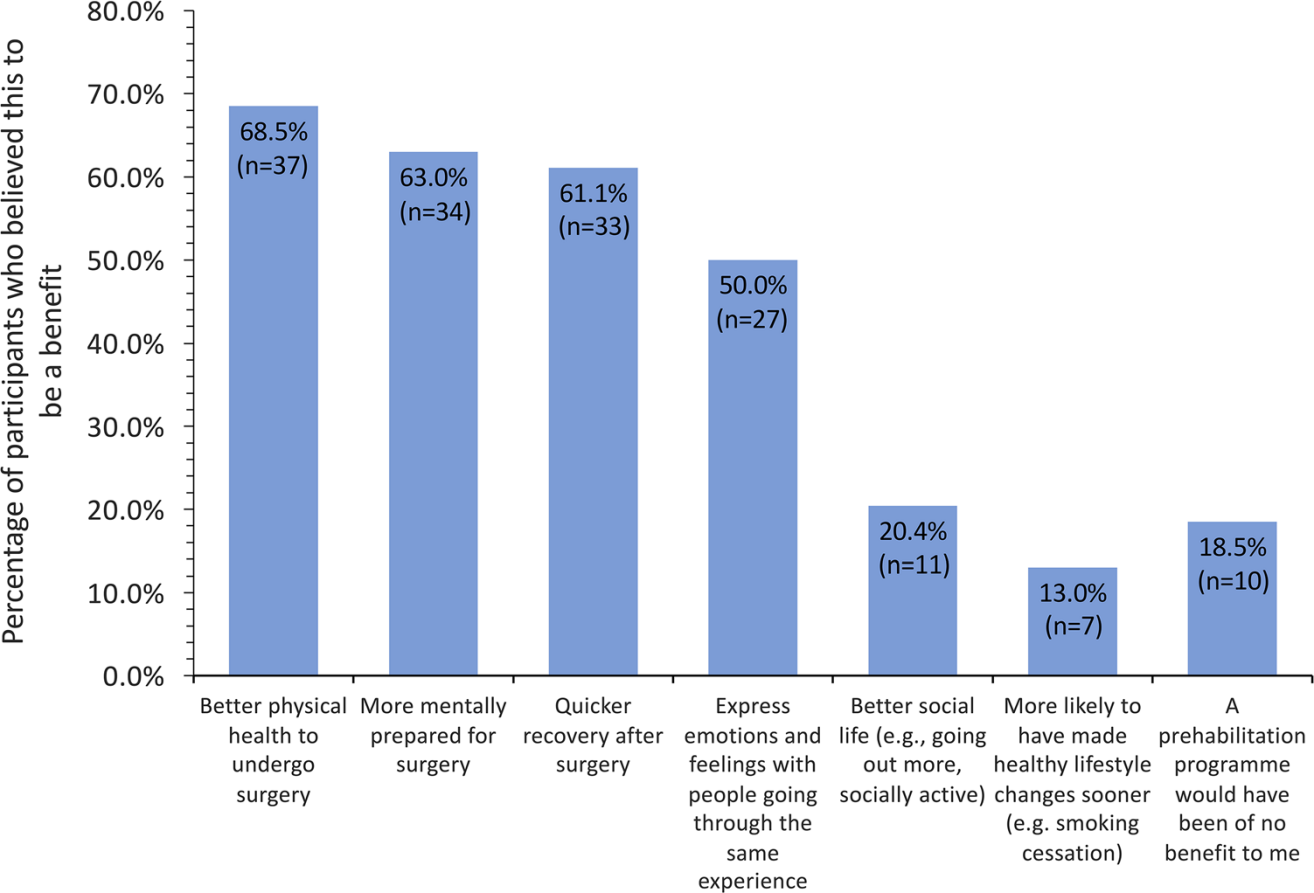
Views of participants in the CARE programme on the potential benefits of a prehabilitation programme before surgery (*n *= 54)

Forty-four (81.5%) participants said they would have joined a prehabilitation programme had it been available before surgery or treatment. The views of the participants on the perceived benefits of prehabilitation are summarised in Fig. 1, with greater than 60% recognising that prehabilitation could have helped them to mentally prepare better for surgery and enhance recovery. However, 10 (18.5%) responders indicated they would have opted not to join a prehabilitation programme, had it been available at the time of diagnosis.

### Design of a prehabilitation programme

**Fig. 2:**
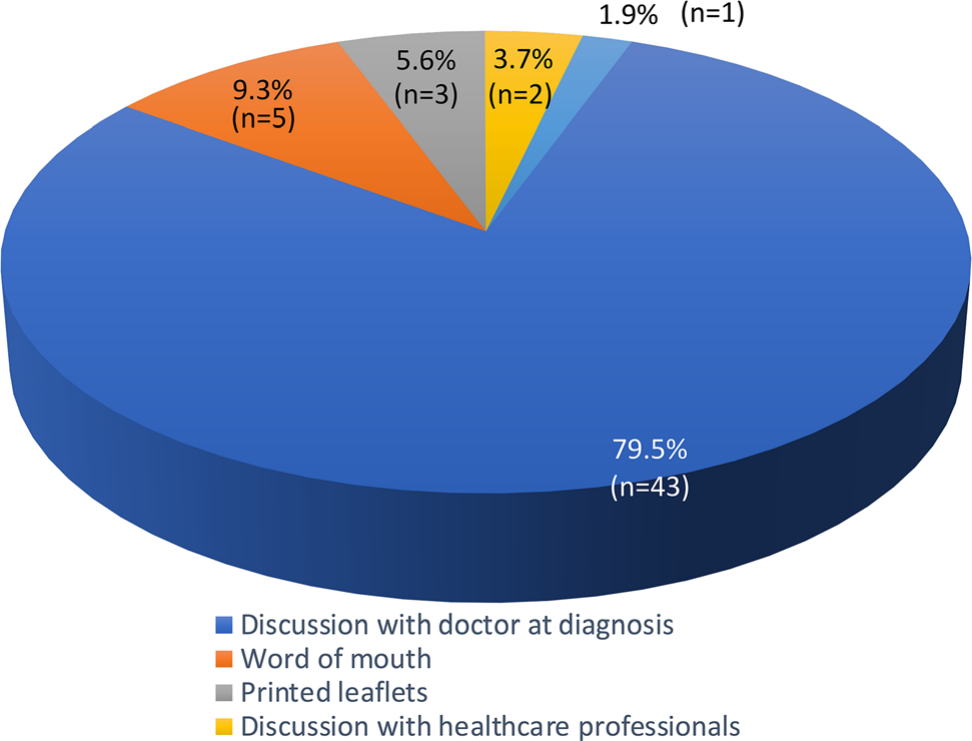
A pie chart illustrating the way participants in the CARE programme would wish to be informed of prehabilitation (*n *= 54)

Most participants (*n *= 43, 79.6%) indicated they would have liked their doctor to have introduced prehabilitation to them at the time of diagnosis. Figure 2 also shows the other preferences of the participants for communication.

Physical exercise was ranked as the most important aspect of prehabilitation by 28 (51.8%) participants, with cardiovascular and running activities considered potentially the most beneficial (Table 2). In addition, 35 (64.8%) participants indicated they would have benefitted from dietary advice. With regards to the frequency of prehabilitation sessions, 26 (48.1%) participants said they would have liked to attend sessions on a weekly basis, whilst 22 (40.7%) said they would prefer them scheduled 2–3 times per week. When asked about their preferred location, 31 (57.4%) respondents said they would like the programme to be based at a centre, while 22 (40.7%) requested a combination of home- and centre-based sessions. Only one participant opted for an in-hospital programme.

**Table 2:** Types of exercise that participants felt would have been of most benefit to them before surgery/treatment

Type of exercise	*n*	%
Cardiovascular (e.g. aerobic exercises—running, walking, and swimming)	25	46.3
Muscular (e.g. weight training and resistance training)	11	20.4
Flexibility (e.g. yoga and pilates)	10	18.5
Balance exercises	8	14.8

**Fig. 3:**
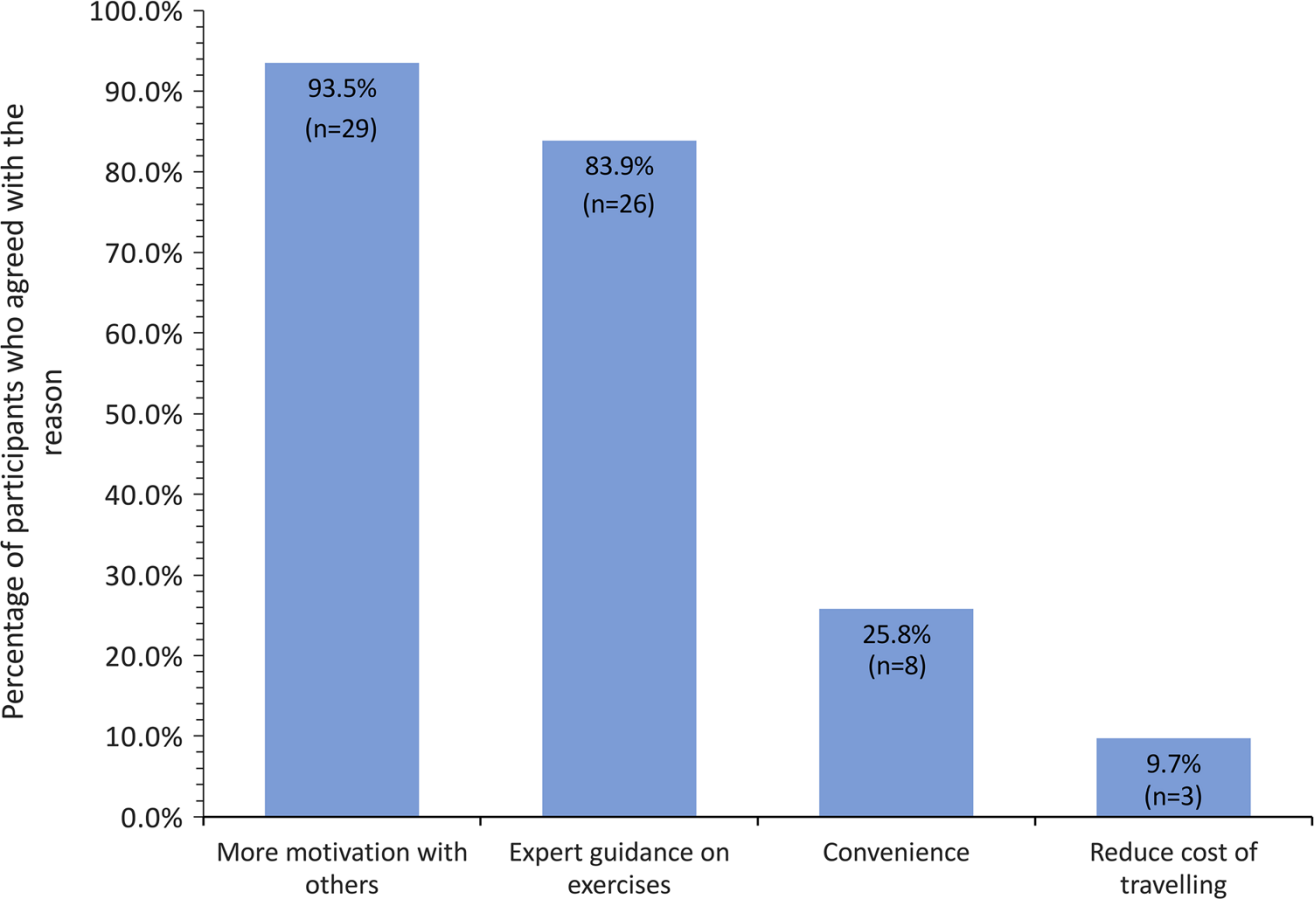
Bar chart of the principal reasons offered by 31 CARE programme participants who expressed a preference for a centre-based prehabilitation regimen

Of the 31 respondents who preferred centre-based sessions, 29 (93.5%) felt they would be more motivated to participate in the activities in the company of others, while 26 (83.9%) felt that they could get better access to expert guidance on exercises (Fig. 3). In contrast, 15 respondents (27.8% of the whole cohort) opted for mixture of both home and centre-based sessions, as this would be reduced travel times and offer greater flexibility in terms of when exercise sessions could be undertaken.

With regards to psychological preparation for treatment, 40 (74.1%) participants said they felt mental wellbeing before surgery was ‘very important’ for a successful outcome, based on quicker recovery time and the potential for fewer complications. In addition, 33 (61.1%) respondents said they would like to have attended counselling classes to manage stress and anxiety prior to surgery, had these been available, with 22 (40.7%) willing to attend weekly sessions and 8 (14.8%) preferring reduced frequency of one every two weeks.

### Thematic analysis

**Fig. 4:**
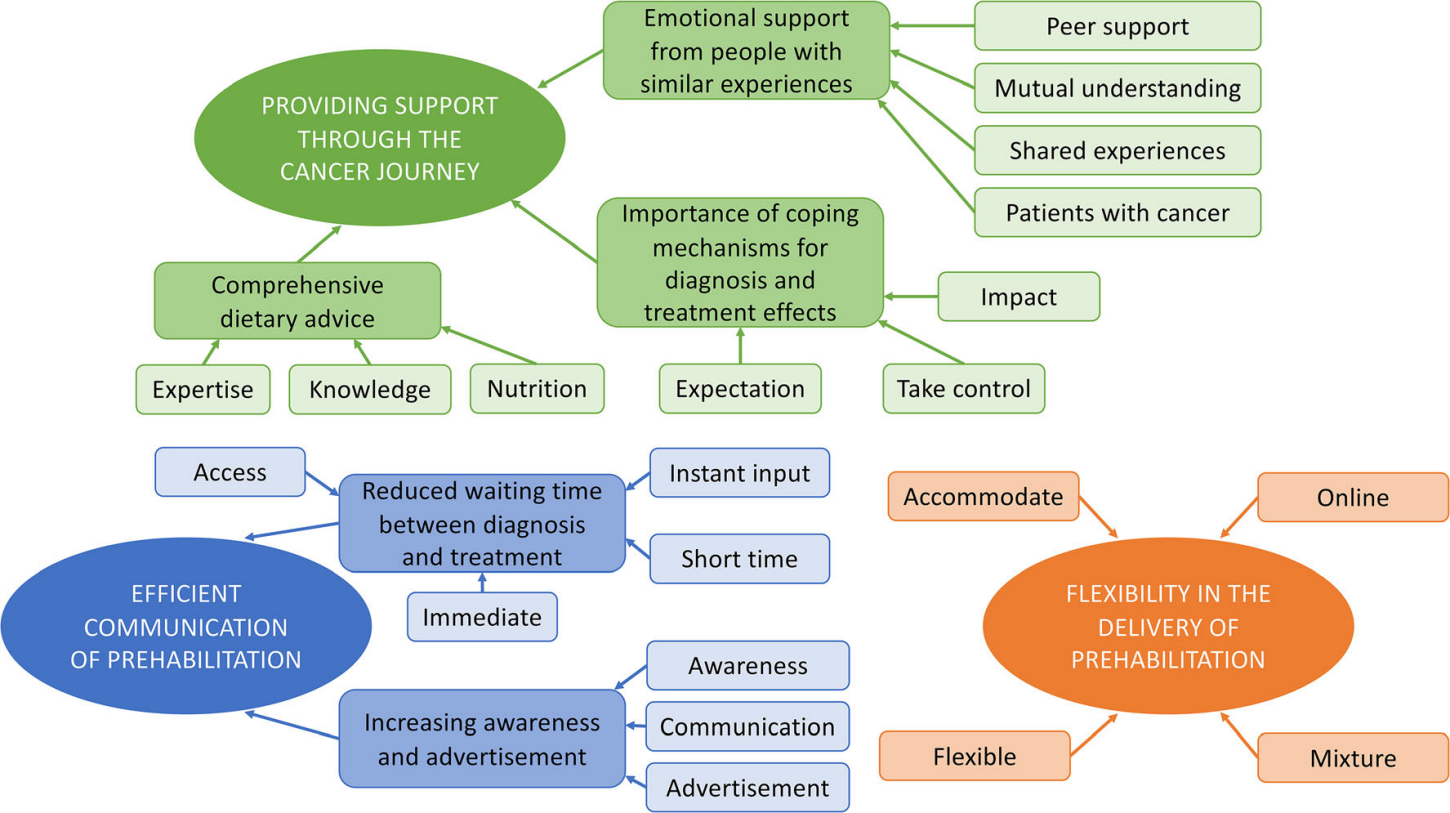
Map of the themes and subthemes in free text comments from CARE programme participants about a prehabilitation programme

Three themes were identified from the 24 free text responses: ‘Support Through The Journey’, ‘Communication’ ‘Flexibility of Programme’ (Fig. 4). Table 3 shows a number of the key text comments related to the ‘Support Through The Journey’ theme that also highlight the importance and need for ‘better dietary advice’.

**Table 3:** Summary of comments made by participants in the thematic analysis of free text responses to questionnaire (*n *= 24)

Themes	Quotes from patients’ free text responses
Improved communication and awareness of such a programme	“Prehabilitation needs to be communicated at diagnosis with the doctor in a clear and simple way to avoid information overload” “[Prehabilitation is a] brilliant idea but needs more advertisement”
The need for a supportive community during the cancer journey	“Meeting and exercise with a group of people starting this frightening journey would have helped me enormously” “Going through cancer has been a lonely experience, I feel I have had little support. The best support has been from other cancer patients” “Being around others who have had similar experiences has been important in providing encouragement to keep going”
Patients want dietary advice to be a key part of their care throughout their journey	“The only advice I was given was to get fat for chemotherapy, no attention to nutrition” “Most dietary advice I've had has not been comprehensive enough” “I wanted to talk to someone who had expertise in diet, particularly probiotics and immunity” “I felt frustrated that the dietary advice I received had amounted to a single worksheet”

Three main themes were identified: improved advertisement of prehabilitation, the need for a supportive community and improved dietary advice

## Discussion

The potential benefits of prehabilitation regimens for patients undergoing surgical procedures and non-surgical treatment for cancer is currently subject to intense research globally, in part because high-quality evidence of benefit remains elusive [3]. This study provides valuable insights into the type of multi-modal programme that may appeal to patients diagnosed with cancer and it also offers options to improve engagement. A unique feature of the cohort participating in this study was that none of the respondents had been given the opportunity to participate in a prehabilitation programme following diagnosis for cancer, but all had engaged with the CARE programme following completion of their treatment for cancer. To ensure potential respondents were informed about prehabilitation we produce a supporting video and directed them to appropriate online sources of information. Respondents expressed a strong willingness to participate in a prehabilitation programme, had it been offered to them after diagnosis. A clear message is that most of the respondents believed it would have helped them to be physically fitter prior to surgery and better prepared mentally for the full treatment pathway. This outcome largely agrees with similar patient-based surveys undertaken by individuals at the start of their treatment for cancer [15–17].

In addition to the physical benefits offered by cardiovascular-based activities in a prehabilitation programme, respondents identified access to professional counselling and peer support from other patients also dealing with a diagnosis of cancer (Fig. 1) as key features. It is worth noting that while this cohort of patients represent only 25% of those originally approached for their views, it is possible that they comprise a subgroup of ex-patients who have remained actively engaged with the community-based CARE programme. This is underlined by the high proportion of respondents in possession of home exercise equipment or considering purchasing such equipment. As such, they recognised the benefits arising from making major lifestyle changes following treatment for cancer and were also well-placed to comment on the potential value of earlier exposure to prehabilitation in the treatment pathway. This committed subgroup of patients, who had successfully completed the treatment pathway, were in effect ‘role models’ and could provide additional support for patients with newly diagnosed cancer. A focus group-based investigation of patients with colorectal cancer noted that many respondents highlighted the need for ‘shared patient experience of surgical journey’ as an important part of a prehabilitation programme [15]. It is noteworthy that some of the written comments of our cohort underscore the above points—“Going through cancer has been a lonely experience, I feel that have a little support. The best support has been from other cancer patients” (Table 3).

With respect to introducing the idea of prehabilitation to patients, it was clear that respondents would prefer to be informed by their doctor at the time of diagnosis, as this is likely to increase adherence to the intervention. This is a similar finding to another patient-based study [17] and underlines the importance of medical recommendation at the beginning of the treatment journey. One possible way of increasing the likelihood of this happening would be better advertising of prehabilitation programmes in the waiting areas of Cancer and Surgery units.

There was a strong preference in our study for centre-based approach for prehabilitation programmes, with respondents arguing that participants would be more motivated to exercise in the company of others. This is a significant finding, particularly as half of the respondents had access to home exercise equipment. Group activities also help patients with similar conditions to offer mutual support and improve psychological health. It is worth noting, however, that several patient-based interview studies undertaken before and during the COVID-19 pandemic have also highlighted home-based prehabilitation exercise regimens as a preferred option, with travel costs and time being major considerations for hospitals with large catchment areas [23–26]. In support of this option, technological developments in telemedicine clearly make home-based prehabilitation programme more manageable, offer better patient access to nutrition advice and allow for adherence to exercise to be better monitored [24]. The catchment area for the Notts County Foundation CARE Programme is relatively small and runs out of three community-based centres roughly 15 miles apart, which may explain the preference for centre-based activities.

While the optimal duration of any prehabilitation programme is still unknown, our study also highlighted for that some participants the interval between diagnosis and surgery may be too short and become a barrier to joining. In fact, nearly half of the respondents who were against taking part in a prehabilitation programme had a 1–2 week wait time for surgery, suggesting that they believed the time was too short to see any real benefit, consistent with other studies [3, 18].

Based on thematic analysis, it is noteworthy that many participants commented on the lack of nutritional support available to them during their treatment. A number of respondents reported that they undertook extensive research into diets that potentially would ‘optimise the immune system’. As noted in other studies [15, 17], our findings underline the potential value of including nutritional support as part of a multi-modal prehabilitation programme and ensuring that patients have better access to support staff.

Interestingly, in two interview-based studies [15, 16] patients highlighted that support of family members was an important factor in enhancing their perioperative experience. There were also the recurrent themes of (i) lack of personalised exercise programmes, (ii) the need for nutrition prescriptions, (iii) the absence of access to shared patient experiences and (iv) the feelings of frustration and anxiety regarding hospital procedures. The latter point included the repetitive gathering of information and poor communication across departments. These gaps in the surgical journey clearly impacted on their ability and motivation to prepare for surgery and rehabilitate after the procedure [15]. Another qualitative study on 16 participants showed that healthcare staff needed to empower patients to ask questions pertaining to their perspectives of “the what, when, where, who, and why of prehabilitation” [18].

### Limitations

Since only a quarter of the patients on the Notts County Foundation CARE Programme database completed the questionnaire, we recognise the possibility that this may have introduced a bias from those who have a strong interest in exercise or prehabilitation. It is also noteworthy that at the time of the study, approximately half of those approached were no longer routinely involved in leisure centre activities. This issue may have been compounded by the COVID-19 pandemic. Nonetheless, it is important to note that the number of participants in this investigation exceeded those in similar studies [15, 16, 18]. To date, only one study looking at patient perspectives on prehabilitation has had managed to recruit more participants [17].

## Conclusion

Participants exhibited a clear interest in prehabilitation for better physical and mental preparedness for surgery, although short wait times between diagnosis and treatment proved to be a barrier. They expressed the need for a focus on physical exercise, counselling to improve mental health and access to personalised nutritional advice in the perioperative period. Centre-based programmes were felt to provide participants with a supportive community and being introduced to the intervention by a doctor at the time of diagnosis could improve compliance. Taken together, our study provides evidence that prehabilitation may further strengthen shared responsibility that the patient may feel during their treatment journey. Future studies should further explore the effectiveness of prehabilitation in the context of the continuum of cancer care by gaining patients’ perspectives on interventions that could lead to improved compliance and outcomes.

### Supplementary Information

Below is the link to the electronic supplementary material.

Supplementary file1 (DOCX 25 KB)

## Data Availability

Data will be available for sharing upon reasonable request from AKJ (mzyaj9@exmail.nottingham.ac.uk).
