# Supplementary material for: Post-Cancer Treatment Reflections by Patients Concerning the Provisions and Support Required for a Prehabilitation Programme
Source: World J Surg. 2023 Sep 12;47(11):2724–32. doi: 10.1007/s00268-023-07170-7 (PMC10545643; doi:10.1007/s00268-023-07170-7)
Supplement: Supplementary file 1 — Supplementary file1 (DOCX 25 KB) [file 268_2023_7170_MOESM1_ESM.docx]

**Appendix 1**

Questionnaire created that was given to CARE patients via email and paper copies were also distributed to obtain patient’s opinions on the concept of prehabilitation

**Prehabilitation Survey: What would help cancer and surgical patients in the future?**

This questionnaire is produced by medical students from the University of Nottingham as part of their degree research project. The aim is to discover the best approach to improve the health of cancer and surgical patients before any treatments (prehabilitation). We want to know your experiences and opinions of what patients want for any future prehabilitation programme.

Your responses will be kept anonymous and we thank you in advance for your help. Please do not enter any identifiable information throughout (e.g., names of people).

1. Do you wish to participate in this survey? By clicking 'yes' you give your consent for us to use your answers in our project.
2. Yes, I give permission for my answers to be used in the research projects
3. No, I do not give my permission

**Demographics**

Age (in years) _________________

Sex (please circle):

1. Male b) Female c) I prefer not to say

Type of cancer: ___________________________________

What treatment did you receive? (check all that applies)

1. Surgery
2. Chemotherapy
3. Radiotherapy
4. Hormone therapy
5. Others please specify __________________
6. None

If you had surgery, how long was your waiting time between the diagnosis and surgical procedure?

1. 1-2 weeks
2. 3-4 weeks
3. 5-6 weeks
4. 7-8 weeks
5. More than 8 weeks
6. I did not have surgery

**About the CARE Programme**

1. When did you join the CARE Programme?
2. Before treatment
3. During treatment
4. After treatment

How many weeks before your treatment did you join the CARE programme? (If not applicable, write N/A)

__________________________ weeks

2) How did you find out about the CARE programme?

- 1. Leaflets in discharge package
  2. Word-of-mouth
  3. Discussion with doctor
  4. Social media (e.g., Facebook, Twitter, etc.)
  5. Others (please specify) ___________________

3) Did you miss any of the CARE sessions out of 12 weeks?

1. None
2. A few (1-2 sessions)
3. About a third (3-4 sessions)
4. About a half (5-6 sessions)
5. Most of them (7-9 sessions)
6. Nearly all of them (10-12 sessions)

4) If you missed sessions, what was the reason?

1. Loss of motivation
2. Transport issues
3. Physical too ill to attend
4. Did not enjoy sessions
5. Couldn’t find time to attend
6. Not applicable as I attended all sessions
7. Others (please specify)

5) Did you have to complete a questionnaire (on your own or with a doctor or nurse) asking questions about your physical or mental health before your cancer treatment?

1. Yes, mental health questionnaire
2. Yes, physical function tests (e.g. 6-minute walking test)
3. Yes, both mental health questionnaire and physical function test
4. No, I was not asked any of this*

*Would you have liked to have been asked questions about your physical and mental health before surgery?

1. Yes, I would have liked to have been asked questions about my physical and mental health
2. No, I would not have liked to have been asked questions about my physical and mental health

6) How has the CARE programme impacted you? (Check all that apply)

1. Better physical health
2. Quicker recovery
3. Improved mental health
4. Improved confidence
5. Long term lifestyle changes (e.g., stopped smoking, stopped drinking alcohol)
6. Introduction to a support community of people having similar treatments
7. Adopted a healthier diet
8. It has had no impact on me
9. Too straining on my body (e.g., caused injury, made me feel tired)
10. Lowered my confidence (e.g., made me feel worse about myself)
11. Others (please specify) _______________________

7) During the process of your journey, how much support did you feel you received from the CARE programme on a scale of 1-5? (1 = not supported, 5 = very supported)

1 2 3 4 5

8) Why do you continue to come to the rehabilitation programme although you have completed 12 sessions of the CARE programme? (circle all that applies)

1. Social reasons (i.e., peer support, making friends who are on the same journey)
2. Physical health reasons (i.e., keeping fit)
3. Mental health reasons (i.e., better psychological support, relieves anxiety and stress)
4. I do not continue with the sessions after completing the CARE programme
5. Others please specify ____________________

9) Did you exercise (any physical activity for at least 15 minutes per time) during the COVID-19 lockdown?

1. Yes, I did*
2. No, I did not

*How many times did you exercise per week?

1. 1-2 times
2. 3-4 times
3. 5-7 times

*Where did you exercise during the COVID-19 lockdown?

1. At home by myself
2. At home via online classes (e.g. Zoom)
3. Outdoor exercises (eg: cycling, trail walking)

**About Prehabilitation Programme**

10) Have you heard of prehabilitation?

1. Yes b. No

11)  Prehabilitation is the physical and mental preparation before a major surgery or cancer therapy (unlike rehabilitation which comes after). The aim is to help improve outcomes (e.g., less complications post-surgery) and reduce the time taken to recover. It is a personalised plan that focuses on exercise, nutrition and mental health of a patient prior to major surgeries or cancer therapies. It can also help patients to make lifestyle changes following their diagnoses.

Now knowing the definition, would you say that you have participated in a prehabilitation program?

1. Yes*
2. No

*What elements of the prehabilitation programme did you participate in? (Circle all that you have participated in prior to the beginning of your cancer therapy)

1. Exercise training
2. Nutritional support (i.e., dietary advice, protein supplementation)
3. Psychological support (i.e., group meetings, wellbeing sessions)
4. Behavioural changes (i.e., counselling for stopping smoking or stopping drinking alcohol)

12) Would you like to have joined a prehabilitation programme if it was available to you before your surgery/treatment?

1. Yes
2. No

13) What would be the main reason for not joining a prehabilitation programme?

1. See no benefits of it
2. Transportation problems
3. Do not have the time for it (e.g., busy schedules)
4. Time between diagnosis to treatment is too short to join to see benefits
5. Mentally not prepared to take part in a programme just after being diagnosed
6. Other (please specify)
7. Not applicable as I would join the programme (N/A)

14) How would you have liked to have been introduced to a prehabilitation programme?

Select one choice

1. Leaflets
2. Discussion with doctor at diagnosis
3. Social Media (e.g., Facebook, Twitter, etc.)
4. Word-of-mouth
5. Others (please specify) _____________

15) How often would you attend sessions in a prehabilitation programme?

1. Once a week
2. 2-3 times a week
3. 4-5 times a week
4. Twice a month
5. Once a month
6. Never

16) How do you think a prehabilitation programme would have benefitted you before your surgery, if at all? (Circle all that applies)

1. Better physical health to undergo surgery
2. More mentally prepared for surgery
3. Quicker recovery after surgery
4. Express emotions and feelings with people going through the same experiences
5. Better social life (e.g., going out more, socially active)
6. More likely to have made lifestyle changes sooner (e.g., stopping smoking, drinking, etc.)
7. Other (please specify) ______________
8. A prehabilitation programme would have been no benefit to me

17) If you were part of a prehabilitation programme, which of these aspects would be most important to you? Rank most important (1) to least important (4)

________ Physical exercise

________ Nutrition

________ Psychological support

________ Behavioural change → advice on stopping smoking, drinking alcohol

18) How important do you feel your mental health is before surgery for a successful outcome e.g., speedier recovery, less complications?

1. Very important
2. Somewhat important
3. Not important
4. My mental health before surgery has no effect on the outcome of my surgery

19) Do you feel like rehabilitation alone is sufficient or both prehabilitation (i.e. before treatment) plus rehabilitation (after treatment) together would be more beneficial?

1. Only rehabilitation would be sufficient
2. Both would be more beneficial

20) Would you prefer the programme to be:

1. Home-based
2. Centre-based programme (e.g., community leisure centre, local gym)
3. In the hospital
4. A mixture of home-based and centre-based programme (e.g., home-based with one centre-based session per week)

Why? (Check all that applies)

1. Convenience (i.e., reduce time to travel, flexibility on time)
2. Reduce the cost of traveling
3. More motivation with others
4. Expert guidance on exercises
5. Other please specify___________

21) Do you have exercise equipment at home?

1. Yes, I have some at home
2. No, I do not have any at home*

Would you buy some exercise equipment for a prehabilitation programme at home?

1. Yes, I would buy some b) No, I would not buy some

22) What type of exercises would have been of most benefit to you before going through surgery/treatment?

1. Cardiovascular (i.e., aerobic exercise - running, walking, swimming)
2. Muscular (i.e., weight training, resistance training)
3. Flexibility (i.e., yoga, pilates)
4. Balance exercises

23) Would you like to have attended counselling or psychological classes to manage stress and anxiety from diagnosis up until the surgery?

1. Yes*
2. No

*How often would you have liked to attend counselling classes from diagnosis up until surgery?

1. Once a week
2. Twice a week
3. Once every 2 weeks
4. Once a month
5. Once every couple of months

24) Do you think you would have benefitted from dietary advice before your surgery/treatment?

1. Yes
2. No

25) How helpful would you have found dietary advice to change your eating habits on a scale of 1-5? (1- not helpful at all, 5 - very helpful)

1 2 3 4 5

26) (Optional) Any comments/ ideas you have for a prehabilitation programme __________________________________________________________________________________________________________________________________________________
